# The British Medical Association

**Published:** 1902-08-09

**Authors:** 


### The Hospital

A Journal of

The Medical Sciences and Hospital Administration.

Vol. XXXII.—No. 828. Edited by Sir Henry Burdett, K.C.B., and Solomon C. Smith, M.D., M.R.C.P. [August 9, 1902.

### The British Medical Association.

The meeting of the British Medical Association at Manchester, which was held last week, appears to have suffered in some degree from the general absorption of the public mind in topics of a totally different order, such as the health of the King, the return of officers and soldiers from South Africa, and the parliamentary wrangles over the Education Bill. Whatever may be the explanation it is at least true that it has attracted less than the customary amount of notice from the non-medical press, and we are even inclined to think that, for some reason or other, it has deserved less. To whatever extent this may be true it is to be regretted, because a great gathering together of medical practitioners in a populous centre is an event which would be utilised, if those concerned in its management were wise, for the establishment of a better understanding than now exists, on the part of the public, of the aims and objects of the profession, of the extent to which the attainment of these aims and objects would be conducive to the public good, and of the agencies and procedures by which they might be attained. There are undoubtedly many points on which the medical profession and the public might understand each other better than they do, and might so understand each other to their reciprocal advantage; and we have often thought that the British Medical Association might with great propriety follow the example of the British Association for the advancement of science, and might make the delivery of an address to the public, by some man of light and leading, a prominent feature of the yearly gathering. Such lectures as some of those which have been delivered at Birmingham by Mr. Priestley Smith, on matters relating to the eyes, or as the *Conferences sur l'Empirisme* of Trousseau, would attract numerous and attentive audiences, and might at least lay the foundation of some knowledge of the objects and methods of the man of science, as distinguished from the objects and methods of the charlatan, whether the latter personage be considered as he exists within the profession, or in his more luxuriant and unchecked growth beyond its ranks. Few things could have been more appropriate, on the present occasion, than the organisation of a complete debate on the question of the prevention of enteric fever in the field; a debate which should have laid under contribution at once the most advanced knowledge of the epidemiologist, and the practical acquaintance of the army surgeon with the requirements and the possibilities of the march, as well as with the amount of co-operation to be expected either from the officers and non-commissioned officers or from the rank and file. The losses from enteric fever in South Africa have been so large, and so widely diffused over all ranks, that the subject has been brought home to the minds of vast numbers of people in this country; and, at the same time, Dr. Leigh Canney has come forward as the advocate of the systematic supply of boiled water to troops, whether in camps or in the field, as a perfectly practicable means of destroying the bacillus in a medium through which it gains frequent access to the human body. In the absence of precise knowledge, as we have formerly pointed out, there must be differences of opinion as to the degree in which water-borne infection may be supplemented by infection conveyed through other channels, such as flies and dust; but there can be no difference as to the certainty that water is the most frequent source of mischief, or that it could be rendered innocuous in the manner proposed. The whole question really turns on the practicability of the scheme in the conditions of actual warfare; and, in the face of this fact, we hear that a War Office Committee is conducting experiments as to the best methods of sterilising water. If the country should be involved in a great war twenty years hence, it is probable that the pigeon-holes of the Office will then be found to contain some memoranda relating to this question. A distinct pronouncement on the subject by the British Medical Association would serve to bring together the forces of the profession and of the survivors of those who have perished from the disease, and thus to create a co-operation between doctors and the public for the attainment of an object which should be equally desired by both, and which might easily lead to similar co operation for other purposes. There is a great opportunity before the Association, and, like other opportunities, if lost it will probably be lost for ever. In that case, and bearing in mind that it has been said that, in this country, politicians "live in bondage to fools," the British soldier, like Charles XII., will for an indefinite time be

> "Condemned a needy suppliant to wait,
> While ladies interpose, and slaves debate."

The leaders of the Association, if they believed in the power of the body over which they preside, and if they were strong enough for the responsibilities imposed upon them, would search for questions on which they might bring precise knowledge to the support of popular feeling, and might so win their way to be listened to on questions in which the public interest is less apparent than the interests of the profession.

